# RET Gene Abnormalities and Thyroid Disease: Who Should be Screened and When

**DOI:** 10.4274/Jcrpe.870

**Published:** 2013-03-01

**Authors:** Behrouz Salehian, Raynald Samoa

**Affiliations:** 1 City of Hope, Department of Diabetes, Endocrine & Metabolism, California, USA

**Keywords:** RET gene, thyroid cancer, screening

## Abstract

Mutations in the RET proto-oncogene have been implicated in the pathogenesis of several forms of medullary thyroid cancer (MTC). Multiple endocrine neoplasia type 2 (MEN-2) is an autosomal dominant syndrome caused by germline activating mutations of the RET proto-oncogene and has been categorized into three distinct clinical forms. MEN-2A is associated with MTC, bilateral pheochromocytoma, and primary hyperparathyroidism. MEN-2B is associated with MTC, bilateral pheochromocytoma, and mucosal neuromas. The rarest clinical form of MEN-2 is familial MTC (FMTC), which is also associated with MTC, but other endocrinopathies are characteristically not present. Each clinical form of MEN-2 results from a specific RET gene mutation, with a strong correlation of phenotype expression with regard to the onset and course of MTC and the presence of other endocrine tumors and a corresponding genotype. Recommendations for screening of RET mutations are necessary as their presence or absence will influence interventional strategies such as the timing of a prophylactic thyroidectomy and extent of surgery. Timing of screenings and development of interventional strategies are extremely important in caring for patients with certain RET mutations as evidence of metastatic MTC has been documented as early as 6 years of age. Interventional strategies should consider the risks of complications of these interventions based on certain characteristics of each individual case such as age of the patient, course of disease in affected family members, and the invasiveness of any proposed surgical procedure.

**Conflict of interest:**None declared.

## INTRODUCTION

The RET proto-oncogene is a member of the tyrosine kinase (TK) superfamily and encodes a cell membrane receptor TK (1). Mutations in the RET proto-oncogene have been implicated in the pathogenesis of several forms of thyroid cancer. Germline mutations of RET are responsible for the development of heritable forms of medullary thyroid carcinoma (MTC), while somatic mutations of this oncogene are found in a significant proportion of sporadic MTCs (2). Further, rearrangements of the RET gene papillary thyroid carcinoma (RET/PTC) are associated with PTC commonly seen in tumors of children and tumors associated with radiation exposure (3). 

**Biological Characteristics of the RET Gene and Protein**

The location of the RET gene was determined to be on chromosome 10q 11.2 in 1985, and the gene subsequently named RET (re-arranged during transfection) after it was found to be rearranged during transfection in 3T3 cell lines with DNA from lymphoma cells (4). RET contains 21 exons and encodes a tyrosine receptor protein that consists of a transmembrane domain, an extracellular domain with a ligand binding site, and an intracellular TK domain (5). 

The natural splicing of the RET gene codes for multiple proteins, including 3 major isoforms. These isoforms, RET 51, RET 49 and RET 9, are differentiated by the number of amino acids at their C-terminal, which are 51, 49 and 9 amino acids, respectively (6). The RET protein has 3 domains, which include an N-terminal extracellular domain that is a ligand for an activator protein called glial cell derived neurotropic factor (GDNF), a hydrophobic transmembrane domain, and an intracellular TK domain (7). The TK domain contains multiple tyrosine residues (16 in RET 9 and 18 in RET 51), two of which, at positions 1019 and 1051, are only present in RET 51.

**Figure 1:**
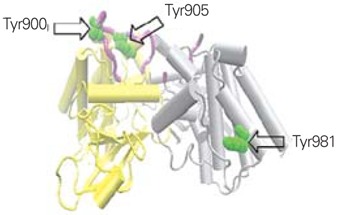
Structure of the RET homodimer. The structure is that of a dimerformed between two protein molecules each spanning from amino acids703-1012 of the RET molecule, covering RET’s intracellular tyrosine kinasedomain. One protein molecule, molecule A, is shown in yellow and theother, molecule B, in grey. The activation loop is shown in purple andselected tyrosine residues in green. Part of the activation loop from moleculeB is absent. (http://en.wikipedia.org/wiki/RET_proto-oncogene)

The RET protein is 170 KDa, present on the cell surface, highly phosphorylated on tyrosine residues, and activated by an endogenous ligand that belongs to the neuronal growth factor family (8) and is structurally depicted in Figure 1. Binding of this ligand, known as GDNF family ligand (GFL), to RET triggers homodimerization of RET and a transformational change in the RET intracytoplasmic domain (1). A set of ligand, receptor and co-receptor (glycosylinsoitol phosphate receptors or GFRs) interactions are necessary for these transformational changes to occur (9). Once GFL binds to the RET receptor, an intracytoplasmic domain within upstream portion of the RET protein is autophosphorylated, which stabilizes the protein, and is necessary for further downstream activity of the RET autophosphorylation cascade. In fact, phosphorylation of Tyr981, as well as of Tyr1015, Tyr1062 and Tyr1096, is important for initiating intracellular signal transduction processes (1).

Known mutations in RET lead to gain of function and to autophosphorylation of tyrosine sites within RET and have been directly implicated in the molecular pathophysiology of multiple endocrine neoplasia type 2 (MEN-2) (10). Greater understanding of the molecular dysfunctions caused by RET gene abnormalities has allowed for the development of screening and treatment recommendations for individual patients. 

**MTC, MEN-2 and Molecular **

**Dysfunction**

Single point mutations in the extracellular domain of the RET proto-oncogene have been implicated in the malignant transformation of cells that originate from the neural crest during embryogenesis (11), such as the parafollicular cells (C-cells) of the thyroid. These point mutations can occur in both germline and somatic cell lines. Germline mutations are associated with disorders that include Hirschsprung disease, which is a congenital absence of the enteric nervous system in the hindgut, and MEN-2. MEN-2 is a dominantly inherited syndrome that includes MTC, which is preceded by C-cell hyperplasia, and the diagnosis of pheochromocytomas, neuroganglionomas, ganglioneuromatosis of the GI tract, paraganglionomas, and hyperparathyroidism within its diagnostic criteria (Table 1) (12). The various tumor components of MEN-2 are all thought to be products of RET proto-oncogene mutation which convert the RET proto-oncogene into a dominant transforming gene, while the non-mutated allele is retained in the tumor (13). Sporadic MTC, on the other hand, is thought to be due to a somatic mutation in the tumor cells (14). 

The age of onset of MTC varies by subtype of MEN-2. MTC typically occurs in early childhood for MEN-2B, predominantly early adulthood for MEN-2A, and middle age for familial MTC (FMTC). Thus, there is a long latency period before any clinical signs of the MEN syndrome develop, which often do not appear until the third or fourth decades of life (15). This provides a large window of time in which to intervene if mutations are detected early and before signs of bilateral and multicentric MTCs develop. This is important because bilateral and multicentric MTCs (16) can be problematic to manage clinically and are the most common cause of death in patients with MEN-2A (17).

**MEN-2A, FMTC and MEN-2B Germline Mutations**

**Figure 2:**
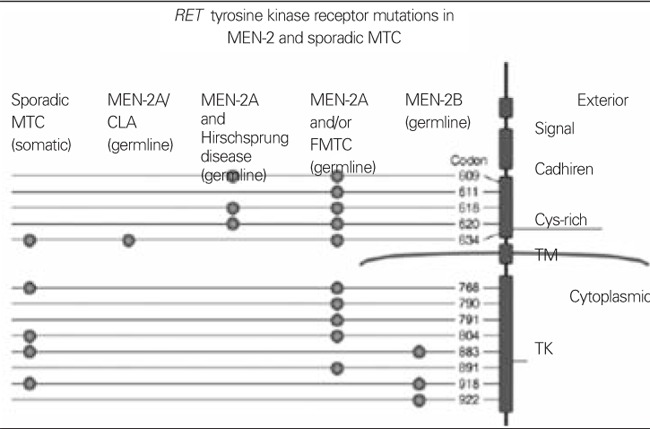
Molecular abnormalities of the RET proto-oncogene in multipleendocrine neoplasia type 2 (MEN-2). Mutations of the RET protooncogenehave been identified in MEN-2A, familial medullary thyroidcarcinoma (FMTC), MEN-2A associated with Hirschsprung's disease,MEN-2A associated with cutaneous lichen amyloidosis (CLA), and assomatic mutations in sporadic MTC. Two regions of the RET tyrosinekinase (TK) are affected. The first is a cysteine-rich extracellular domain(Cys-Rich) important for dimerization of the RET receptor (codons 609,611, 618, 620, 634). Mutations of individual cysteines at these codonscause RET dimerization, activation, autophosphorylation, andtransformation. Mutations of the second region, the intracellular TKdomain, which involves codons 768, 790, 791, 804, 883, 891, 918, and922 cause activation, autophosphorylation, and transformation. A role forthe cadherin-like region (Cadherin) has not been defined, although it canbe used as a marker in blood in future studies. Image fromwww.elsevierimages.com/image/endocrine.htm; with permission

The current recommendations for surgical intervention in any familial thyroid malignancy arose from observations of the relationship of RET gene abnormalities with the clinical pattern of these malignancies, which strongly suggested that the mutated gene was the controlling factor for the clinical expression of these disorders. Molecular abnormalities of the RET proto-oncogene and the associated disorder of MEN-2 and sporadic MTC have been listed in Figure 2.

Development of FMTC is preceded by C-cell hyperplasia (CCH), which is characterized by high numbers of C-cells within a follicular space forming a nodular hyperplasia. The diagnosis of CCH requires the presence of at least 50 C-cells immunostained with calcitonin per lower power magnification (x100) microscopic field in adults (18). In children, C-cell population reference ranges are not well established (19). FMTC is usually bilateral and multicentric, and the presence of CCH is considered a genetically determined precursor to the malignant phase of the disease. 

Because MTCs must be treated surgically, it is imperative to rule out other MEN-2A-associated conditions that may lead to an adverse surgical outcome if not properly treated preoperatively. For example, pheochromocytomas are present in half of patients with MEN-2A (20), and surgical management of a pheochromocytoma should take priority over a thyroid resection in patients with MEN-2A to avoid a potential catastrophic hypertensive crisis (21). While benign functional parathyroid adenomas are present in approximately 10 to 20 percent of patients with MEN-2A (20) and should be diagnosed prior to surgery in patients with MEN-2A to coordinate any necessary parathyroid surgery with any thyroid resection (21). 

As for MEN-2B, this disorder arises from a single missense mutation of paternal origin and is typically an aggressive disease, but the risk of aggressive disease can be stratified by the codons affected. MTC can occur by the first year of age and includes nodal metastasis. The documentation that metastatic MTC can arise before 6 years of age raises concern about waiting until children are older to consider a prophylactic thyroidectomy in patients that have these mutations (22,23). 

Less is known about FMTC, rarer variant of MEN-2A, which occurs in 10-15% of patients with MTC. Other manifestations of MEN-2A rarely develop in FMTC and are easily mistaken for sporadic MTC. In fact, one study reported that among 729 patients who appeared to have sporadic MTC and who were studied over a 10-year period, RET genetic screening identified an unsuspected germline RET mutation in 6.5% of these patients (47 out of 729 patients) (24), which would generally reclassify these patients as having FMTC.

**RET Gene Abnormalities in MTC**

Germline mutation of the RET proto-oncogene commonly resides in codons 609, 611, 618, 620, 630, and 634 (2,24,25,26,27), as well as codons 768, 790, 791, 804, 883, 891 and 918 (26) within the intracellular domain (Table 2). These codons are encoded by exons 10-16. The most prevalent germline mutation seen in patients with MEN-2A is within codon 634. It is detected in 80-90 percent of MEN-2A cases and, in 50% of these cases, consists of a change of a cysteine to an arginine residue. Most cases of MEN-2B have a mutation in codon 918, although rare instances of mutations in codons 833 and 922 have also been reported. These 3 codons represent 5% of all hereditary MTC (28). In FMTC, mutation of codon 620 was detected in 95% of all cases and in 6 to 8% of patients with MEN-2. Most of these mutations involve the extracellular cysteine residue, which allows RET to activate phosphorylation of tyrosine residues in downstream proteins such as PLC gamma, P38 MAPK, and JNK. However, in all cases of MEN-2B and in certain cases of FMTC, the intracellular domain of RET is affected as well.

There is a high correlation between mutation of the RET gene and whether affected individuals clinically present as MEN-2A or MEN-2B. Therefore, during management of MTC, sequencing of the entire RET coding region should be requested only if exon-specific testing is negative. Sequence analysis of exons 10, 11, and 13 to 16 has been reported to be abnormal in 98% of cases of MEN-2A. In addition, single point mutations that change methionine 918 to threonine or alanine 883 to phenylalanine are the usual genotype abnormalities detected in patients with MEN-2B. Should sequence analysis fail to detect the usual abnormalities, then testing for a point mutation at position 804 should be performed followed by sequencing of the entire RET coding region. For FMTC, sequence analysis of exons 10, 11, and 13 to 16 has detected mutations in 95% of cases. Since MTC and FMTC have similar clinical presentations, people with isolated MTC should be offered germline testing for FMTC (27). 

**Correlation Between Genotype and Tumor Phenotypes**

Because there are strong correlations between RET activating mutations and their corresponding phenotypes, genetic testing is an invaluable tool for early diagnosis and intervention (27). Unfortunately, due to the potentially long latency period before development of disease, genetic testing is limited in advising clinicians and patients on optimal timing of these interventions, such as surgery. However, in any given family, the relative course of an effect of a RET germline mutation in one family member will resonate more frequently in other members of the family and can be used to assist in making clinical decisions in an asymptomatic affected family member. There is limited evidence that polymorphisms in RET or in other components of the RET signaling system, such as GFR alpha-1, may influence the age of onset of a RET mutation associated tumor phenotype or have a traceable modifier effect on disease expression (28,29). 

**Screening Recommendations**

The outcome of patients with newly diagnosed MTC is largely inferior to that of family members who were positively screened for a RET germline mutation (23). Patients who were screened had a 94% rate of cure as compared to non-screened patients (50% 5-year survival), therefore, it is important to screen family members of patients with MTC.

Patients who are diagnosed with MTC should undergo germline mutation analysis to detect mutations in the RET proto-oncogene. If a RET proto-oncogene mutation is confirmed, subsequent work-up should include an evaluation for a possible pheochromocytoma and hyperparathyroidism. Because these mutant alleles involve autosomal dominant transmission, 50% of these individuals’ offspring and kindred might be affected, which has prompted experts to recommend that family members of patients with MEN-2A be referred for genetic counseling. Due to the early onset of MTC associated with certain RET mutations, it has been suggested that the optimal time to determine the genetic risk of individuals with positive family histories of MTC is prenatally. Analysis of DNA extracted from fetal cells obtained by amniocentesis performed between the 15th to 18th weeks of pregnancy or by chorionic villus biopsy between the 10th to 12th weeks of pregnancy facilitates planning and management of the affected fetus after birth. Pre-implantation genetic diagnosis is available as well. The potential risks to offspring and reproductive options of young adults that are affected or at high risk should be addressed via consultations with a geneticist. Patients with MEN-2 who are pregnant can benefit from placental biopsy or amniocentesis, and, if concern arises about the risk of a neonatal patient, the diagnosis can be established while the child is still in the nursery through blood DNA analysis (27).

For kin of affected persons who have a positive germline mutation, the specific type of germline mutation will direct the optimal timing for surgery (codon directed). Patients who are at highest risk are those who have at least one parent that has developed MEN-2B, and for which the codon involved is 883, 918 (95% of MEN-2B) or 922. Recently, tandem mutations of codons 805, 806 and 904 in cis configuration have also been reported in individuals with MEN-2B.

MEN-2B patients often do not have a family history of MTC and, 50% of the time, have a de novo mutation. Notably, their offspring who have a germline mutation often develop aggressive disease with early onset that requires very early attention (25). 

**Timing of Prophylactic Thyroidectomy**

In 2009, the American Thyroid Association (ATA) published consensus guidelines for the timing of prophylactic thyroidectomy for the treatment of hereditary MTC (30). The recent classification of ATA-D MEN-2 equates to the previous Highest Risk Category. The expected aggressive disease course, which requires multiple surgical resections over a patient’s lifetime, necessitates a thyroidectomy in the first year of life for patients in this high-risk category. Affected children may present with more advanced disease that includes lymph node metastasis in the central compartment. Therefore, a total thyroidectomy with central compartment lymph node dissection should be undertaken in such children at the age of 6 months, along with sampling of level II to V of the neck, and, if necessary, extensive non-disfiguring lymph node dissection should be performed if metastatic disease is found.

Consideration of a total thyroidectomy before 5 years of age is recommended for patients in the High Risk Category or ATA B with a RET mutation in codons 609, 611, 618, 620 630, or 631. It is recommended that patients classified as ATA C with a RET mutation in codon 634 have a total thyroidectomy performed before they reach 5 years of age (Table 3) (30). There is no consensus for central neck compartment lymph node dissection (31), although proponents of this approach argue that it reduces the likelihood to need to reoperate in the future and that long-term complications of a central neck dissection can be minimized (32). On the other hand, critics of routine central neck dissection argue that nodal disease is very rare in children less than 10 years of age (17).

Patients classified in the lower risk category of ATA A with MEN-2A or FMTC, that involve distal codons (768, 790, 791, 804, or 891), carry an intermediary risk, suggesting that the decision to proceed to a thyroidectomy can be delayed until certain signs arise. One approach is to wait until calcitonin levels begin to increase consistently, while another is to perform total thyroidectomy before the patient is 5 years old. Some experts recommend that patients should be referred for surgery by the age of 10 years, regardless of the calcitonin levels. Close surveillance that includes routinely checking calcitonin levels and/or referring for pentagastrin/calcium stimulation tests should be conducted to ensure the earliest possible detection of MTC. Stimulated (by calcium or pentagastrin) serum calcitonin levels greater than 100 pg/mL have been recommended as the cutoff for referring patients for surgical intervention (33,34).

A somatic mutation of the RET gene is detected in 25% of cases of the sporadic form of MTC. However, in 6% of these cases, a point mutation in codons 609, 611, 618, 620, or 630 is detected, suggesting that these cases are actually germline mutations. Therefore, attention must be paid to avoid missing any germline mutations, and the referral of family members of patients with tumors that have germline mutations for appropriate screening should be considered. 

The RET proto-oncogene is a dramatic example of the impact molecular analysis can have on a patient’s diagnosis and management at an early stage of life. An advantage of genetic testing, as compared to biochemical assays, is that it requires only a single blood test for DNA analysis and is tempered by the mild side effects of a venipuncture. Another advantage is that it provides information long before biochemical changes such as CCH occur, which by then may be too late to change the clinical course of the disease. Yet, another advantage is that RET proto-oncogene mutations usually remain consistent within a family, which suggests that once a mutation is identified, at-risk family members, needs to be screened only once in their lifetime, compared to annual biochemical screening. Family members with a mutation would benefit from total thyroidectomy, whereas family members that lack a mutation (and their descendants) require no further testing. However, it is important to note there is no evidence that the presence of persistent and/or recurrent MTC is dependent on the specific codon mutation. The goal of operative treatment would then become to perform the thyroidectomy before MTC develops or while it is still confined to the gland (36). Immediate normalization of serum calcitonin post-operatively has a favorable prognosis, whereas a progressive increase in serum calcitonin levels after surgery needs to be reevaluated for another possible operation. Studies have shown that the biochemical cure rate is higher (44%) when serum calcitonin before surgery is less than 1000 pg/mL, compared to levels above 1000 (1%) (35). 

**Complications Associated with Thyroidectomy**

The decision to proceed to surgical resection should be considered after an assessment of the risk of possible complications from the surgery. A thyroidectomy with central compartment resection involves a total resection of both thyroid lobes, including the isthmus, removal of neck fat, and central compartment lymph node dissection. The risks of complications are higher in children than adults, and the thyroidectomy should be performed in centers by qualified personnel with sufficient experience.

Complications that affect the vocal cords include recurrent laryngeal nerve palsy, which is reported to occur ipalsy that requires life-long intubation and tracheostomy. Complications involving the parathyroid glands.The decision to proceed to surgical resection should be considered after an assessment of the risk of possible complications from the surgery. A thyroidectomy with central compartment resection involves a total resection of both thyroid lobes, including the isthmus, removal of neck fat, and central compartment lymph node dissection. The risks of complications are higher in children than adults, and the thyroidectomy should be episodeperformed in centers by qualified personnel with n up to 5 percent of children that receive thyroidectomy, unilateral recurrent laryngeal nerve damage, and bilateral recurrent laryngeal nerve include hypoparathyroidism, which is rare but requires treatment with calcium and active vitamin D3 (1.25 dihydroxy vitamin D3) to prevent hypocalcemic s that include muscle spasms, tetany, and seizure disorders. Treatment is also geared to prevent chronic effects of hypocalcemia such as basal ganglia calcification, cataracts, and, rarely, congestive heart failure. Management of hypoparathyroidism is particularly difficult and may affect the quality of life of affected children. Intraoperative death is extremely rare. The risks of these complications increase with each reoperation and should be thoroughly discussed with patients that require multiple operations. 

Surgery performed on patients who have an undiagnosed pheochromocytoma and who have not been properly prepared preoperatively may be fatal. Therefore, it is recommended that plasma levels be measured and/or an analysis of a 24-hour urine collection be made to detect normetanephrines and metanephrines prior to any surgery of a patient with MTC. While preparing patients for a total thyroidectomy, counseling should also include the need for life-long thyroid hormone replacement therapy and the follow-up that this entails. 

**Papillary Thyroid Carcinoma**

RET rearrangements have been reported to be the second most common genetic abnormality found in PTCs. It is estimated that RET/PTC is found in an average of 20% of adults with sporadic PTC, although prevalence estimates from 12 countries range from 0% to 57% (36). RET rearrangements are estimated to exist in 50 to 60% of PTCs in children and in 60 to 70% of radiation-induced PTCs (37). 

RET is highly expressed in parafollicular C-cells but not in follicular cells. RET/PTC rearrangements can activate expression of the RET proto-oncogene. During these rearrangements, the 3’ portion of the RET gene, which frequently involves the 3.0-kb intron 11, is fused to the 5’ portion of various unrelated genes. Eleven forms of RET/PTC arrangements have been reported to date, all identified by the fusion of the RET gene to a different partner (Table 4) (36,37,38). RET/PTC1 and RET/PTC3 are the most common rearrangements found in PTC. Although several studies have reported that RET rearrangements are associated with PTCs that generally do not progress to poorly differentiated or anaplastic carcinomas, different rearrangements have been reported to be associated with different types of tumors (39). The RET/PTC1 rearrangement is more common in classic PTC, papillary microcarcinomas, and the diffuse sclerosing variant subtypes. Conversely, RET/PTC3 is more often associated with radiation-induced papillary tumors that occur after exposure to high levels of radioactivity, tumors that are of short latency, are more aggressive and are of the solid variant subtype (40,41). 

RET/PTC has been found to transform thyroid cells in culture and gives rise to thyroid carcinomas in transgenic mice. Several studies have suggested that the proximity of RET and the other fused genes in the identified RET/PTC rearrangements may allow for a single radiation tract to produce a double strand break in each gene at identical sites in the nucleus, thus generating these rearrangements (42). As a consequence of the rearrangements, RET comes under the control of the fused heterologous genes (listed in Table 4). The chimeric RET is then ubiquitously expressed in neoplastic thyroid follicular cells, a cell type in which it usually is not expressed. When the RET/PTC rearrangements are translated into fusion proteins, the coiled domains of the translocated amino terminal regions allow the RET/PTC proteins to form dimers, which then allows RET to become activated independent of ligand binding, which is a necessary step for the neoplastic transformation of thyroid follicular cells (43). Although tyrosines are autophosphorylated in wild-type RET in a ligand-dependent manner, the RET/PTC protein dimers lead to constitutive kinase activity and RET autophosphorylation, which results in amplified stimulation of the pathway that mediates the biologic effects of wild-type RET (44,45,46). 

Now that RET/PTC has been identified as a marker for PTC and can be accurately detected by RT-PCR and combined immunochemistry, potential applications for RET/PTC detection are being proposed. RET/PTC identification is currently being evaluated to assist with the evaluation of thyroid lesions that are difficult to diagnose due to ambiguous histological features. In a small clinical study, identification of RET/PTC rearrangements refined the diagnosis of 60% of cases that would have otherwise have been considered indeterminate and 33% of specimens that were considered insufficient for cytological diagnosis (47). Further, the same study reported that no false positive cases were identified, which strongly suggests that RET/PTC rearrangements have high specificity as a marker of PTC. Currently, clinical guidelines do not include RET/PTC identification in the work-up of thyroid nodules and/or the management of PTC. 

RET/PTC rearrangements still code for the TK domain of the RET receptor and enable the RET/PTC oncoprotein to bind to a transforming protein called SHC, which has been implicated in activation of the mitogen-activated protein kinase signaling pathway (48). This has provided a target for molecular therapy in radioiodine-refractory and unresectable differentiated PTC. A recent study compared the effects of four TK inhibitors (XL184, vandetanib, sunitinib, and axitinib) on cell proliferation and RET inhibition and looked for mutation specificity using cell lines that harbored a MEN-2A mutation (MTC-TT), a MEN-2B mutation (MZ-CRC-1), or a RET/PTC rearrangement (TPC-1). All four TK inhibitors were shown to reduce cell proliferation in vitro to some extent. However, XL184 was the most efficient inhibitor for MEN-2A- and PTC-derived cell lines (49). There are few reports describing the efficacy of TK inhibitors in the management of PTC. Two phase II studies evaluated the effects of sorafenib in the treatment of differentiated thyroid cancer. One study of 41 PTC patients confirmed partial response in 15% of patients and stable disease was described in another 61%. For PTC patients whose cancer had not previously been treated with chemotherapy, median progression-free survival was 16 months (50). The other study aimed to evaluate the effect of 26 weeks of sorafenib therapy on radioiodine uptake and tumor response in 32 patients with progressive, radioiodine-negative differentiated thyroid cancer. Twenty-five percent of these patients had a partial response, 34% had stable disease, 22% had progressive disease, and 19% were nonevaluable. The median progression-free survival was 58 weeks (51). In a recent retrospective series, sorafenib therapy was associated with prolongation of median progression-free survival by at least one year, compared with patients’ rate of disease progression before initiation of therapy (52). A randomized, placebo-controlled phase III study of sorafenib as first-line therapy for progressive metastatic differentiated thyroid cancer is currently being conducted (53). Lenvatinib, a new potent thyrosine kinase inhibitor and vascular endothelial growth factor 2 and 3 receptor EGFR1 inhibitor, has been shown to yield promising results (54) and personal observation) in management of advanced thyroid cancers.

**Summary**

Germline activating mutations of the RET proto-oncogene have a strong penetrance of MTC, which provides an opportunity to identify patients at high risk for metastatic MTC. RET mutation screening provides a unique model for early prevention of metastic MTC in patients with MEN-2 and asymptomatic carriers of RET mutations. Early identification also prompts the screening of commonly associated endocrinopathies that need to be addressed prior to any surgical intervention to reduce the likelihood of preventable adverse complications and the need for subsequent surgeries. RET mutation provides a useful resource in caring for patients, but treatment plans for patients with MEN-2A must also consider the risk and benefits of complications from surgical interventions that are often influenced by the characteristics of patients, such as their age and the course of MEN-2A in affected relatives.

## Figures and Tables

**Table 1:**
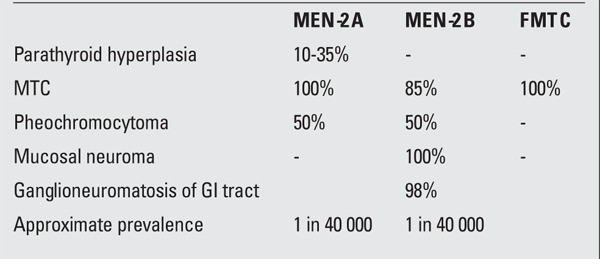
Features of multiple endocrine neoplasia type 2 (MEN-2)syndromes and of familial medullary thyroid carcinoma (FMTC)

**Table 2:**
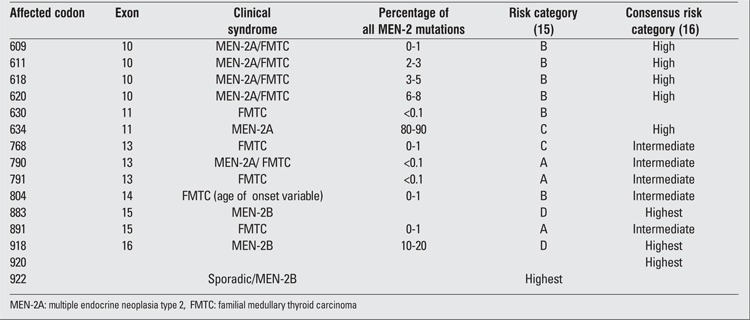
Germline mutations of the RET proto-oncogene in MEN-2A ([ref:24]24[/ref])

**Table 3:**
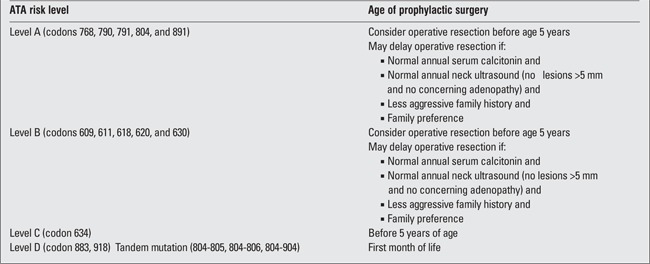
American Thyroid Association (ATA) risk level and timing of prophylactic thyroidectomy in multiple endocrine neoplasia type 2 (MEN-2A)

**Table 4:**
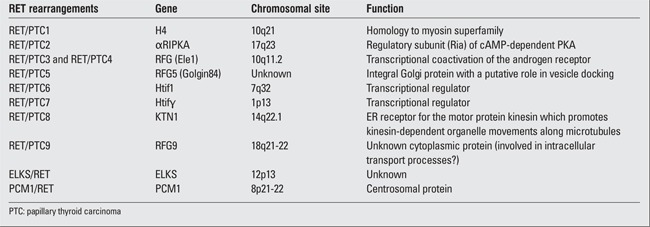
RET/PTC rearrangements ([ref:36]36[/ref])
